# Executive Functioning in Adolescents with Chronic Musculoskeletal Pain

**DOI:** 10.3390/children7120273

**Published:** 2020-12-04

**Authors:** Kristen E. Jastrowski Mano, Emily A. Beckmann, Lauren M. Fussner, Susmita Kashikar-Zuck

**Affiliations:** 1 Department of Psychology, University of Cincinnati, Cincinnati, OH 45221, USA; beckmaea@mail.uc.edu; 2 Department of Child and Adolescent Psychiatry and Behavioral Sciences, Children’s Hospital of Philadelphia, Philadelphia, PA 19104, USA; fussnerl@chop.edu; 3 Division of Behavioral Medicine and Clinical Psychology, Cincinnati Children’s Hospital and Medical Center, Cincinnati, OH 45221, USA; susmita.kashikar-zuck@cchmc.org

**Keywords:** executive functioning, pediatric, chronic pain, adolescents, functional impairment, working memory, inhibition, cognitive flexibility

## Abstract

Adolescents with chronic pain often suffer significant impairment in physical, emotional, and social domains. Surprisingly little is known about executive functioning (EF) in youth with chronic pain or how EF deficits may contribute to functional impairment. Study participants included 60 adolescents between the ages of 12 and 17 years (*M* = 14.57). Thirty participants with chronic musculoskeletal pain and 30 age- and gender-matched healthy controls were recruited from a large Midwestern children’s hospital in the United States. Participants completed the Behavior Rating Inventory of Executive Functioning (BRIEF-2) as well as multiple measures of functional impairment across key domains: school, social, emotional (anxiety, depression), and physical. Adolescents with chronic musculoskeletal pain reported significantly greater EF impairment compared to healthy age- and gender-matched peers. Clinically elevated risk levels of impairment were reported across all aspects of EF, with many adolescents in the chronic pain group scoring above the clinical risk cut off for working memory (52%), inhibition (45%), and cognitive flexibility (38%). EF was also significantly related to functional impairment across all domains. Findings suggest that EF may have an impact across several critical domains of functioning for youth with chronic pain.

## 1. Introduction

Pediatric chronic pain is a critically important health problem [1] associated with profound impairments in several areas of daily living, including school [2,3,4,5], peer relationships [6], and emotional functioning [7,8,9]. One critical mechanism in relation to functional impairment that has yet to be thoroughly investigated in pediatric chronic pain is executive functioning (EF), which refers broadly to a set of cognitive processes that are central to self-regulatory functions (i.e., processes involved in controlling one’s behavior, emotions, and thoughts in the pursuit of a goal) [10,11].

This gap in knowledge exists despite the documented deleterious influence of pain on attention [12,13,14] and accumulating evidence suggesting that EF negatively impacts functioning and exacerbates pain-related disability [15,16]. Indeed, adults with chronic often report feeling more functionally incapacitated by cognitive dysfunction than by pain symptoms [17,18,19,20]. EF mechanisms have also been well documented in other pediatric populations. For example, among youth with type I diabetes, EF is viewed as a vulnerability factor linked to self-management, such that EF deficits are related to poorer adherence, which, in turn, predicts poorer glycemic control [21,22]. EF has also been shown to be a robust predictor of response to treatment [23] and health-related quality of life in pediatric epilepsy [24] and sickle cell disease [23].

**Figure 1:**
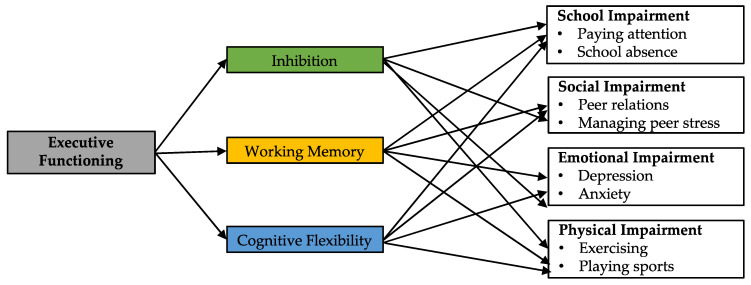
Model predicting core EF mechanisms linked to each key area of functional impairment in pediatric chronic pain.

The relationship between EF and functional impairment in youth is a complex interplay of factors that change with developmental level [10]. Why might EF be a particularly important mechanism related to functional impairment in pediatric chronic pain? One reason is that when adolescents experience pain, the degree to which they can inhibit attentional capture by pain, re-orient attentional resources toward something else, and subsequently maintain the [non-pain] orientation of attentional resources largely depends on EF [12,13,14,25]. Consequently, the interruptive effect of pain on EF likely has negative effects on multiple functioning domains: academic, social, emotional, and physical. See Figure 1. In terms of academic impairment, adolescents with chronic pain report significant difficulty paying attention in school, as well as frequently missing school and falling behind academically [2,3,4,5]. In the context of social functioning, EF deficits have been identified as a critical mechanism predicting the onset of depression in the face of peer stress [26]. This is highly relevant given the increased prevalence of both peer difficulties [6] and depressive symptoms [7,8] in pediatric chronic pain. Overall, deficits in EF likely prevent youth from demonstrating effective responses to stress (academic-, peer-, and pain-related stress), leading to increased stress and risk for emotional problems.

Theoretical and empirical research agrees that inhibition, working memory, and cognitive flexibility are three core components of EF [27,28,29]; and further, pain has been linked to notable impacts on each of these domains [20,30,31,32,33,34,35,36,37,38]. Inhibition refers to the ability to control attention in order to respond to the demands of a particular task rather than respond impulsively [27]. Working memory involves the maintenance and manipulation of information in mind for a short period of time [39]. Cognitive flexibility involves the ability to switch attention to a different task or to a different mindset, and to be mentally flexible to solve problems [27,29]. These three cognitive abilities are included in the “unity and diversity” model of executive functions [29] and are often referred to as core executive “building blocks” underlying more complex executive functions such as planning or problem solving. This framework has been particularly useful when studying EF in children [40,41].

### Current Study

Despite convincing evidence pointing toward the impact of EF deficits on functional impairment in both adult chronic pain and pediatric health populations [15,16,17,18,19,20,21,22,23,24], literature assessing EF among pediatric patients with chronic pain remains scarce [42,43]. A comprehensive picture of which EF domains are vulnerable in pediatric chronic pain and the degree to which EF may be linked to functional impairment is needed. Clinically, this would strengthen and clarify treatment goals and expand intervention options. We hypothesized that youth with chronic musculoskeletal pain would report worse EF—particularly, inhibition, working memory, and cognitive flexibility—relative to healthy age- and gender-matched peers. We also tested the associations between EF and multiple domains (school, social, emotional, and physical) of functional impairment.

## 2. Materials and Methods

### 2.1. Participants

Participants included 60 adolescents between the ages of 12 and 17 years (*M* = 14.57, *SD* = 1.51). Thirty participants had a primary complaint of localized or widespread musculoskeletal pain (including, but not limited to, complex regional pain syndrome, juvenile fibromyalgia, or idiopathic musculoskeletal pain syndromes) lasting 3 months or longer, diagnosed by a pediatric pain physician or pediatric rheumatologist. Participants in the pain group were recruited from outpatient pediatric pain and rheumatology clinics at a large Midwestern children’s hospital in the United States. Thirty age- and gender-matched healthy controls were recruited using flyers posted on the hospital website and email distribution lists. Participants in the healthy control group had no current or history of chronic pain.

All participants were required to be English-language speaking with no documentation of developmental delay, disease-related pain (e.g., sickle cell disease, inflammatory bowel disease, systemic lupus erythematosus), attention-deficit/hyperactivity disorder (ADHD), or untreated major psychiatric condition (e.g., bipolar disorder, psychosis). The sample included more female participants (66% female) and the majority of participants (86%) identified as White and Non-Hispanic; 8% identified as Black and 3% participants identified as “other” or chose not to disclose their race. Average household income ranged from less than $25,000 to greater than $150,000, with 22% of participants reporting average income greater than $150,000.

### 2.2. Procedure

The study was conducted in accordance with the Declaration of Helsinki, and the protocol was approved by the Institutional Review Board of Cincinnati Children’s Hospital Medical Center (2018–2343). Participants and their primary caregivers completed written informed assent and consent for inclusion before they participated in the study. Interested participants contacted the study coordinator by phone to learn more about the nature of the study and eligibility criteria. Survey measures took approximately 30 min to complete using REDCap, a secure, web-based software platform. Participants were given the option to complete online self-report measures in a designated hospital space or at home. Participants were required to complete measures within one week of receiving the survey link and participants were unable to pause survey completion. After completion of study measures, participants received a $20 gift card.

### 2.3. Measures

#### 2.3.1. Demographics

Participants self-reported their age, grade, race, ethnicity, and average household income. Participants in the pain group also reported primary pain location, duration and intensity. Specifically, participants provided ratings of their current, average, and worst pain intensity on a 0–10 cm visual analogue scale (VAS), anchored by “no pain” and “pain as bad as it can be.” The VAS is one of the most widely used scales for pain assessment and has been validated for use with children over the age of 5 years [44].

#### 2.3.2. Executive Functioning

The Behavior Rating Inventory of Executive Functioning, Second Edition, Self-Report Version (BRIEF-2) is a 55-item inventory assessing youth’s perception of their own executive functions, and purposeful, goal-directed, and problem-solving behavior in their daily life [45]. Items are scored on a 3-point Likert scale ranging from 0 (never) to 2 (often). The BRIEF-2 yields seven subscales including inhibit, self-monitor, shift, emotional control, task completion, working memory, and plan/organize, three composite indices including the Behavior Regulation Index (BRI), Emotion Regulation Index (ERI), and Cognitive Regulation Index (CRI) as well as a total Global Executive Composite score. The present study focused on the inhibit (inhibition), working memory, and Shift (cognitive flexibility) subscales given extant literature identifying these as key EF domains [27,28,29] that appear particularly salient in chronic pain [20,30,31,32,33,34,35,36,37,38]. Raw scores are converted into standardized *T* scores with a mean of 50 and standard deviation of 10. *T* scores between 60 and 64 are considered mildly elevated, between 65 and 69 moderately elevated or borderline clinically significant, and greater than 70 clinically significant. Internal consistency was excellent for the Global Executive Composite (*α* = 0.90), and the inhibit (*α* = 0.95), Shift (*α* = 0.95) and working memory (*α* = 0.98) subscales.

#### 2.3.3. Physical Impairment

The Functional Disability Inventory (FDI) [46] assessed participant’s difficulty completing physical activities across home, school, recreational, and social settings (e.g., walking upstairs, completing activities in gym class). Fifteen items are scored on a 5-point Likert scale ranging from 0 (no trouble) to 4 (impossible) and summed to create a total score ranging from 0 to 60, with higher scores indicating greater disability in physical functioning. The FDI has been validated for use with adolescents with chronic pain [47]. Internal consistency in the current study was excellent (*α* = 0.97).

#### 2.3.4. Academic Impairment

Participants self-reported their academic attendance (i.e., missing full days, arriving late, and leaving early), academic performance, and accommodations (e.g., modified coursework, extra time on assignments, tutoring). Sample items include, “How many full school days have you missed in the last month (i.e., out of the last 20 school days)?” and “What are your average grades currently?” Research suggests high correlations between self-reported grades and performance reports [48].

#### 2.3.5. Emotional Impairment

Participants completed two self-report measures assessing emotional functioning, the Screen for Child Anxiety Related Emotional Disorders-Child Report (SCARED) [49] and the Center for Epidemiological Studies Depression Scale for Children (CES-DC) [50]. The SCARED is a 41-item youth report measure assessing a wide range of anxiety symptoms including generalized worry, panic, social anxiety, separation anxiety, and school avoidance. Items are scored on 3-point Likert scale ranging from 0 (not true or hardly ever true) to 2 (very true or often true) and summed to create a total score ranging from 0 to 82. Total scores greater than or equal to 25 are indicative of at least moderate anxiety. The SCARED has been validated for use with adolescents with chronic pain [51]. Internal consistency in the current study was excellent (*α* = 0.91). The CES-DC is a 20-item self-report inventory assessing depressive symptoms. Items are scored on a 4-point Likert scale ranging from 0 (not at all) to 3 (a lot) and summed to create a total score ranging from 0 to 60. A cut-off score greater than or equal to 15 is indicative of significant depressive symptoms [50]. Internal consistency in the current study was good (*α* = 0.85).

### 2.4. Data Analysis

Analyses were conducted using SPSS Version 27. Independent (2-tailed) *t*-tests were used to test the hypothesis that adolescents with chronic pain would endorse significantly worse inhibition, cognitive flexibility, and working memory on the BRIEF-2 compared to age- and gender-matched healthy controls. To determine associations among key domains of EF (BRIEF inhibition, cognitive flexibility, and working memory) and functional impairment (physical, academic, social-emotional), bivariate Pearson correlations (*r*) were calculated. Hierarchical multiple regression analyses were run to determine the proportion of variance in functional impairment scores explained by executive functioning (BRIEF-2 total scores) above and beyond variance explained by average pain intensity. In each regression analysis, BRIEF-2 total scores and pain intensity were the predictors and one functional impairment measure (FDI, Academic Impairment, CES-D, SCARED) was the criterion.

## 3. Results

### 3.1. Participant Characteristics

The majority of adolescents with chronic pain reported multisite pain lasting greater than 24 months (56%); average pain intensity was moderate (*M* = 6, *SD* = 2.2). The most common location of pain included leg/knee (32%), back (30%), joint (27%), and neck pain (23%). No statistically significant differences were found between the chronic pain group and healthy control group on household income or race (*p* values < 0.05).

### 3.2. Executive Functioning in Adolescents with Chronic Pain versus Healthy Controls

Adolescents with chronic musculoskeletal pain reported statistically significantly greater EF impairment compared to healthy age- and gender-matched controls. The mean raw scores and *T* scores for the BRIEF-2 inhibition [*t*(58) = −2.35, *p* < 0.05; Cohen’s *d* = 0.65], Shift [*t*(58) = −2.45, *p* < 0.05; Cohen’s *d* = 0.80], and working memory [*t*(58) = −3.09, *p* < 0.01; Cohen’s *d* = 0.91] subscales, as well as total Global Executive Composite scores, are presented in Table 1.

Between 38% (cognitive flexibility) and 52% (working memory) of adolescents with chronic pain reported clinically elevated EF on the BRIEF-2. The relative percentages of participants in each group (chronic pain and healthy control) who scored in the clinically significant range (*T*-scores greater than 70) on the BRIEF-2 can be found in Table 2.

### 3.3. Associations between EF and Functional Impairment in Adolescents with Chronic Musculoskeletal Pain

Greater EF impairment (inhibition, cognitive flexibility, and working memory) was significantly associated with worse physical impairment (FDI: *r* ranged from 0.46 to 0.57, all *p* < 0.01), academic impairment (*r* ranged from 0.56 to 0.61, all *p* < 0.01), depression symptoms (CES-DC: *r* ranged from 0.63 to 0.71, all *p* < 0.01), and anxiety symptoms (SCARED: *r* ranged from 0.47 to 0.70, all *p* < 0.01). Bivariate correlations between all BRIEF-2 subscales and measures of functional impairment are presented in Table 3 (physical impairment, academic impairment, and depression symptoms) and Table 4 (anxiety symptoms).

Hierarchical regression analyses revealed that average pain intensity contributed significantly to physical impairment (as measured by the FDI), *F* (1,29) = 13.05, *p <* 0.01, and accounted for 32% of the variance in physical impairment. Adding BRIEF-2 scores to the model explained an additional 10% of variance and this change in *R2* was significant, *F* (2,28) = 4.07, *p* < 0.05, *β* = 0.32. In all other hierarchical regression models, average pain intensity was not a significant predictor of functional impairment, whereas executive functioning (BRIEF-2 Total scores) explained a significant amount of incremental variance in academic impairment (32% (school attendance) to 36% (grades)); depression symptoms (45% (CES-D)), and anxiety symptoms (52% (SCARED)) above and beyond the variance explained by pain intensity (*β* = 0.56 to 0.77, all *p*-values < 0.001).

## 4. Discussion

Adolescents with chronic musculoskeletal pain reported significantly worse executive functioning (EF) compared to healthy age- and gender-matched peers. Clinically elevated levels of impairment were reported, and consistent with previous research [20,43,52], the domains of working memory and inhibition emerged as areas of concern. Indeed, over half of youth with chronic pain demonstrated scores on the BRIEF working memory subscale that were above the clinical cut off. Furthermore, EF was significantly related to functional impairment across all assessed domains: physical, academic, and emotional. This suggests that executive functioning deficits may have a far-reaching impact on youth with chronic pain.

Pain intensity did not contribute significantly to academic or emotional (depression and anxiety) impairment. Rather, the relationship between EF and these domains of functional impairment was significant—in terms of bivariate correlations and incremental variance—whereas pain intensity played a relatively minimal role. This is consistent with previous research that found pain intensity to be a non-significant factor when predicting school impairment and quality of life in children and adolescents with chronic pain [53,54,55,56]. Not surprisingly, in contrast to academic and emotional functioning, pain intensity was significantly associated with physical impairment. However, EF explained a significant amount of incremental variance above and beyond pain intensity in the prediction of physical impairment. Taken together, it seems that pain characteristics do not invariably and directly predict functional impairment, but rather, complex transactions among cognitive (i.e., executive functioning) and affective (e.g., pain catastrophizing) factors are also involved.

It is evident from the range of scores on the BRIEF that adolescents with chronic pain do not universally experience significant difficulties with EF. As a group, their average BRIEF score indicated “average” to “mildly elevated” [45] EF impairment. However, a clearer picture emerged upon closer inspection of within-group variability in EF scores. Specifically, a sizeable subset of adolescents with chronic pain (approximately 40–50%) appear to experience more severe symptoms in terms of global EF as well as in one or more of the “building blocks” of EF—namely, inhibition, cognitive flexibility, and working memory. We would argue that the adolescents with chronic pain who experience clinically elevated EF impairment would be the subset at greatest risk for functional impairment.

Given the importance of cognitive processes in treatment adherence and response [21,22,23], chronic pain patients with EF deficits may benefit from targeted EF training at pre-intervention and/or adapted intervention approaches that are less cognitively demanding (e.g., less taxing on working memory). Further, a chronic pain patient’s ability to benefit from pain self-management interventions—cognitive behavioral, lifestyle modifications, and/or exercise-based therapies—is intimately related to EF. For example, distraction techniques—a common clinical intervention used in pain treatment [57]—demand the inhibition of attentional capture by pain and re-orientation of attentional resources toward something other than concurrent pain [12,13,14]. Thus, in order for intervention approaches to be effective for patients with chronic pain and EF deficits, select strategies may need to be tailored, for example, through use of external tracking or reminder systems and inclusion of caregiver support.

EF is a domain of cognitive functioning that is also uniquely crucial for adherence to medical regimens [21,22] and planning activities based on ones’ current physical condition [58], such as deciding whether or not to go to school or work when experiencing pain. Consequently, for adolescents with chronic pain, difficulties with EF may have myriad negative effects on day-to-day functioning. In the academic realm, chronic pain patients often report significant difficulty paying attention in school (e.g., trouble focusing on what the teacher is saying, or on what they are reading), periods of missed school, and falling behind academically [3,4,5,59,60]. The degree to which EF deficits contribute to these common school-related difficulties is a fundamental area for future research.

The present study focused on self-reported day-to-day impact of executive functioning, which is both clinically relevant and a common means of assessing EF in clinical settings [28,42,45,52,58,61,62]. However, given the multifaceted nature of executive functioning and the many ways each domain can be assessed, much more work is needed to identify the key areas of concern for adolescents with chronic pain. Multimethod, comprehensive assessment of EF will be critical to establish which key areas are most impaired in pediatric chronic pain. Furthermore, it is likely that some EF measurement approaches (e.g., self-report and performance-based measures) will not be highly correlated with one another given that they reflect different aspects of EF [61,62].

The relationship between EF and functional impairment may also vary across chronic pain conditions. The present study focused on chronic musculoskeletal pain. However, patients with different primary pain conditions, such as functional abdominal pain or headache, may show unique patterns of EF deficits, or perhaps, different associations between EF and functional impairment. Additionally, the majority of participants in our sample identified as White and female, thus limiting the generalizability of results. Finally, the mechanisms underlying the association between EF and functional impairment cannot be discerned from this cross-sectional study. Longitudinal work is needed to discern the temporal association between the development of chronic pain and EF deficits, as well as whether EF impairment represents a transdiagnostic risk factor for chronic pain and emotional comorbidities (e.g., anxiety and depression symptoms). Such investigations are needed to establish the developmental course of EF and functional impairment trajectories over time.

## 5. Conclusions

Improved understanding of EF in pediatric chronic pain is needed in order to establish the EF mechanisms involved in functional impairment, adherence, and intervention outcomes, as well as bring us closer to implementing novel interventions that target EF. The relationship between EF and functional impairment in youth likely involves a complex interplay of factors that change over development [40,56].

## Figures and Tables

**Table 1:** BRIEF-2 mean raw scores and *T*-scores, adolescents with chronic musculoskeletal pain (*n* = 30) compared to age- and gender-matched controls (*n* = 30).

	*M* ^1^	*SD* ^2^	*T* (Range) ^3^
**Inhibition** *			
Chronic Pain	13.52	4.21	58.90 (40–86)
Healthy Control	11.28	2.51	51.25 (37–72)
**Cognitive Flexibility**^4,^*			
Chronic Pain	13.97	4.02	58.66 (39–85)
Healthy Control	11.14	2.96	49.57 (37–73)
**Working Memory** **			
Chronic Pain	14.55	4.28	60.90 (41–88)
Healthy Control	11.10	3.23	49.43 (38–72)
**Global Executive Functioning** *			
Chronic Pain	93.48	31.66	50.82 (41–90)
Healthy Control	76.68	27.21	57.31 (37–74)

* = *p* < 0.05. ** = *p* < 0.01. ^1^ = Mean BRIEF-2 raw subscale score; ^2^ = Standard deviation; ^3^ = Standardized *T*-scores; ^4^ = BRIEF-2 Shift subscale.

**Table 2:** Percentage of chronic pain (*n* = 30) versus healthy control participants (*n* = 30) in the clinically significant range on the BRIEF-2.

	Percentage Scoring in Clinically Significant Range (*T* > 70)
**Inhibition**	
Chronic Pain	45%
Healthy Control	14%
**Cognitive Flexibility** ^1^	
Chronic Pain	38%
Healthy Control	14%
**Working Memory**	
Chronic Pain	52%
Healthy Control	7%
**Global**	
Chronic Pain	48%
Healthy Control	14%

^1^ = BRIEF-2 Shift subscale.

**Table 3:** Bivariate correlations between BRIEF-2 subscales and physical impairment, academic impairment and depression symptoms (*n* = 30).

BRIEF-2	Functional Disability Inventory	Academic Impairment ^1^	CES-DC ^2^
Inhibition	0.46 **	0.56 **	0.63 **
Working Memory	0.53 **	0.61 **	0.66 **
Cognitive Flexibility ^3^	0.57 **	0.60 **	0.71 **
Task Completion	0.59 **	0.56 **	0.62 **
Self-Monitoring	0.36 **	0.33	0.51 **
Emotional Control	0.46 **	0.54 **	0.65 **
Planning and Organization	0.43 **	0.45 *	0.67 **

* = *p* < 0.05. ** = *p* < 0.01. ^1^ = VAS of perceived academic performance (grades); ^2^ = Center for Epidemiological Studies Depression Scale for Children (CES-DC); ^3^ = BRIEF-2 Shift subscale.

**Table 4:** Bivariate correlations between BRIEF-2 and SCARED anxiety subscale scores (*n* = 30).

BRIEF-2	Panic/Somatic	Generalized Anxiety	Separation Anxiety	School Avoidance	Social Anxiety
Inhibition	0.63 **	0.47 **	0.52 **	0.50 **	0.58 **
Working Memory	0.62 **	0.51 **	0.59 **	0.67 **	0.62 **
Cognitive Flexibility ^1^	0.69 **	0.70 **	0.72 **	0.71 **	0.64 **
Task Completion	0.56 **	0.49 **	0.54 **	0.62 **	0.65 **
Self-Monitoring	0.55 **	0.29 **	0.48 **	0.41 **	0.55 **
Emotional Control	0.65 **	0.62 **	0.62 **	0.71 **	0.49 **
Planning/Organization	0.60 **	0.54 **	0.59 **	0.62 **	0.58 **

** = *p* < 0.01. ^1^ = BRIEF-2 Shift subscale.
